# Does Polychronicity Undermine Procrastination Behavior Through ICTs? Insights From Multi-Level Modeling

**DOI:** 10.3389/fpsyg.2021.733574

**Published:** 2021-10-01

**Authors:** Tao Xiaolong, Nida Gull, Zubair Akram, Muhammad Asghar, Zhang Jianmin

**Affiliations:** ^1^School of Business Administration and Tourism Management, Yunnan University, Kunming, China; ^2^School of Economics and Management, Yanshan University, Qinhuangdao, China; ^3^Hangzhou College of Commerce, Zhejiang Gongshang University, Hangzhou, China; ^4^Suleman Dawood School of Business, Lahore University of Management Sciences, Lahore, Pakistan

**Keywords:** multitasking, ICTs, team cohesiveness, procrastination behavior, role overload

## Abstract

Information and communication technologies (ICTs) are widely used in developing nations as a dynamic solution for socio-economic development. Pakistan has seen a rapid increase in the use of ICTs during the previous decade. The purpose of this study is to examine how polychronicity affects procrastination behavior when it is influenced by ICTs. According to this study, individuals are described as a dynamic and destructive kind of self-regulation failure in ICTs. Procrastination is a behavior that prevents emerging economies from growing from developing countries. We researched the group-level polychronicity influence of the individual behavior and the mechanism of procrastination from a team-level perspective of worker behavior. This study data collected 231 workers from 76 groups working in ICTs in Pakistan. The results revealed that the group polychronicity and the behavior of group members were positively linked by taking the work overload as a mediator. Moreover, group cohesiveness moderates the role between polychronicity and work overload diminishing the mediation procession between-group polychronicity and individual procrastination. The practical importance of this study is to understand the causes of procrastination, and how to decrease this obstacle to a fairer workplace. It also helps to decide the professional route that is most suited to personality characteristics.

## Introduction

The world's information and communication technologies (ICTs) are continually developing (Kandemir, 2014), and letting the technology-use abilities of an individual erode may affect the personal, professional, and social life of that individual (Reinecke et al., 2018). Individuals engage in consecutive or multiple activities at the same time, moving their time between producing and ingesting action by their incomes and social positions (Carrier et al., 2009). However, with the advances in ICTs, the ability to conduct several activities concurrently has increased in the current organizational culture. Many scholars have increased their focus on the impact of multitasking, which explains why people engage in multiple activities sequentially (Dux et al., 2009; Grawitch and Barber, 2013). Aral et al. (2007) describe the terminology used in research studies, including the terms polychronicity, time use preference, task switching, multitasking skills, instantaneous actions, intersecting activities, simultaneous activities, and at the same time tasks. According to Aral et al. (2012), multitasking is mainly done for two reasons: (a) to do tasks more efficiently and (b) to make primary activities more satisfying. They believe that the previous shows the time constraints, whereas the latter increases the benefit gained from main tasks (Junco, 2012). The type of multitasking activity depends on the primary task, and the secondary time uses of travel are the subject of this study (Spink et al., 2002; Ozmutlu et al., 2003).

Individuals have developed new behaviors due to the creation of new ICTs, such as multitasking behavior is described as performing numerous tasks simultaneously (Spink and Park, 2005; Peng and Kamil, 2018). For the last two decades, academic researchers have examined multitasking when at least one of the activities involves ICTs (Lay and Schouwenburg, 1993; Spink et al., 2002; Ozmutlu et al., 2003). The activities may be contemporaneous, indicating simultaneous exposure and action (e.g., driving while listening to the radio), or they may involve frequent motor and attentional switching (e.g., checking a phone while reading a book; Kamble et al., 2014). Multitasking with ICTs is particularly prevalent in societies with a high level of ICTs access and ownership (Zhijie et al., 2019b). These countries, which are generally described as “modern industrial,” “contemporary,” and “highly prosperous,” also have a larger proportion of older people in their demographics and aging rates (Asghar et al., 2020). Procrastination is the action of laying off what we know we should do. At times, our prevention techniques may be rather inventive (He, 2017). Procrastination is best defined as the action of postponing a task that was initially scheduled despite anticipating to be worse off as a result of the delay (Pachler et al., 2018). All conceptualizations of procrastination behavior acknowledge the necessity of postponing, delaying, or deferring a job or choice, consistent with the Latin roots of the term, pro meaning “forth, out, or in favor of” and ante meaning “of tomorrow” (Emerson, 2015; Rebetez et al., 2016). Procrastination is also defined as the deliberate postponement or avoidance of a planned or scheduled job without justification (Reinecke et al., 2018).

Teams differ in the level of cohesiveness (Tziner and Vardi, 1983). The cohesiveness of a team refers to how individuals are drawn to and motivated to stay on the team (Wendt et al., 2009). Team cohesiveness occurs when team members believe their team will assist them in achieving their need for affiliation or status and achieving a common goal (Shin and Park, 2009; Matt Graham and Jones, 2019). As a result, sociologists have classified team cohesiveness into two categories: socio-emotional cohesiveness and instrumental cohesiveness (Bozanta et al., 2016). Socio-emotional cohesion is a sense of belonging that develops when individuals experience emotional fulfillment due to team participation. Instrumental cohesion is a sense of belonging that develops when team members are mutually reliant on one another and believe they could not accomplish the goals of the team working separately (Bozanta et al., 2016; Matt Graham and Jones, 2019).

Although multitasking behaviors of ICTs have been extensively studied at the individual level (Aral et al., 2007), few studies have examined multitasking behaviors of ICTs in groups or teams (Aral et al., 2012). To our knowledge, this is the first study to examine the impact of team multitasking behavior on team member's procrastination behavior (Rebetez et al., 2016). How multitasking activity, which regularly increases work burden, is absorbed into everyday routines of employees whose mental capabilities begin to deteriorate (Carrier et al., 2009). Furthermore, ICTs researchers have confronted the difficulty of developing adequate methodologies for examining complicated patterns of ICTs usage during group-level multitasking (Bozanta et al., 2016). Self-reporting methods are most suitable in such populations due to the increased likelihood of intellectual bias and the rising disintegration of ICTs usage, which is difficult to monitor and recall in a questionnaire (Kleinman, 2010). As a result, it is critical to establish proper methodologies for examining ICTs patterns in this group-level multitasking. These approaches must generate high-quality data and be acceptable to team-level study participants (Colom et al., 2010; Asghar et al., 2018).

In this study, we evaluate the effects of team-level multitasking behavior on procrastination of individual members, with work overload and team cohesiveness acting as mediators and moderators, respectively, in ICTs companies in Pakistan. Participants in this study expressed their opinions and impressions regarding multitasking activities, stating which ones they would be most comfortable with. Our findings illuminate the role of traditional and new ICTs software house employees, most of whom were socioeconomic and cultural members. These findings can be used to inform the development and implementation of future research strategies aimed at reducing employee procrastination. In addition, the current study establishes a foundation for creating methodological recommendations for assessing complicated multitasking behaviors among employees of ICT software houses in Pakistan.

Considering the “double-edged sword effect” of multitasking trends and the possible impact of contextual factors on employee behavior (Mattarelli et al., 2015), the multitasking trend of teams of employees may affect their procrastination behaviors (Singleton, 2019; Kokoç, 2021). However, there is less evidence of research that has been done to link multitasking trends to procrastination. The multitasking trend of the team indicates that the group expects its members to perform multiple tasks simultaneously while also switching between these tasks (Grawitch and Barber, 2013). The single trend reveals that the team wants employees to focus on a particular task and complete each task in order. This feature influences many individual behaviors as an aspect of group culture (Robinson and Kalafatis, 2017). However, few studies have correlated the time characteristics of such teams with procrastination. Therefore, it is indispensable to study procrastination behavior from the perspective of team-level multitasking (Korabik et al., 2017).

## Literature Review and Hypothesis

### Multitasking and Procrastination Behavior

Information and communication technology is a condensation that indicates “Information and Technology of Communication” (Spink et al., 2002). ICT is an inclusive word that refers to all technological advancements that enable the control and transmission of all digital information (Ozmutlu et al., 2003). ICTs encompass all fields of computerized development that exist today to assist individuals, businesses, and organizations. It is tough to represent ICTs since they move at such a quick pace. It is a concern for limiting, recovering, controlling, and transmitting computerized information (Spink and Park, 2005). The ICTs may be defined as the processing and communication agencies and highlights that support instructing, learning, and the breadth of organizational activities in various ways (Dux et al., 2009).

As part of restoration efforts in the IT sector, it is necessary to increase the quality of human resources. One of the important restorations is to train the interns to become persistent workers. The requirement to prepare the IT sector to become long-term employees is critical because the world is currently characterized by vulnerability and rapid change (Aral et al., 2012). Under such cases, a person capable of developing into a permanent employee will learn to adapt to fragility and rapid change (Kandemir, 2014). It is undeniable that social networking and ICTs for organizational reform are being effectively used by practitioners and researchers today as rings of globalization. A social network is a member of the ICTs family in a variety of ways, most notably in terms of its use for academic purposes, communication, and other facets of human endeavor (Aral et al., 2007; Dux et al., 2009).

When given the type of service of social networking and ICTs in media technology, the two concepts are closely tied (Aral et al., 2012). This is especially true when considering the role of each in the area of communication and networking about organizational structure, which may entail the prediction of employee procrastination (Zhijie et al., 2019a). Let us examine the difficulties associated with each. Social networking enables users to exchange ideas, digital photographs and videos, postings, and notify others about online or real-world activities and events (Gull et al., 2021). The online enables people to connect with others who reside in various areas, from inside a city to across the globe. Members may be able to message any other member, depending on the social networking platform (Košíková et al., 2020). In some other cases, members can contact anybody with whom they have a connection, and then anyone with whom they share a connection, and so on (Zhijie et al., 2021).

Information and communication technologies are frequently used to connect audiovisual, telephone, and communications systems *via* a unified infrastructure or connection system (Xiaolong et al., 2021). There are significant economic incentives to integrate the telephone and computer networks into a single unified system of cabling, signal delivery, and administration (Aral et al., 2007; Zhijie et al., 2019a). ICT is an umbrella word that refers to any kind of communication, including radio, television, mobile phones, computer and network hardware, and satellite systems, as well as the numerous services and appliances that accompany them, such as video conferencing and distance learning (Tkiouat et al., 2021; Xiaolong et al., 2021). As a result, it is obvious that social networking is an essential element of ICTs, and that ICTs plays a role in delivering excellent humanistic services, including educational services at various levels, which may help forecast the procrastination of source employee (Chen et al., 2021; Toyama and Hayashi, 2021). Employees who spend excessive time on nonorganizational ICT activities run the danger of procrastinating, resulting in poor performance despite the organizational benefits of ICTs (le Roux et al., 2021).

Procrastination is the action of delaying the least important activities in exchange for more urgent tasks (Svartdal and Steel, 2017) or doing more enjoyable things in substitute of less important things, therefore procrastinating imminent tasks (Gupta et al., 2012). To be classed as procrastination, the conduct must be counterproductive, unnecessary, and delayed. Similarly, it voluntarily postpones a chosen course of action despite anticipating a negative outcome (Rebetez et al., 2014). The application of procrastination to the field of education qualifies members as procrastinators, a term that refers to actions that are delayed (Rebetez et al., 2016). While there is no commonly accepted definition, individual procrastination could be stated as the procrastination of organizational objectives to the point that optimal performance becomes very improbable, resulting in psychological suffering (Mohammed and Nadkarni, 2014; Li et al., 2017).

Robinson and Kalafatis (2017) have continued work for understanding the increased attention to the concept of multitasking. Therefore, certain temporal characteristics at the group level multitasking may affect the procrastination behaviors of employees, including the trend of team multitasking (Rebetez et al., 2016). The multitasking tendency of the team is that the team level supervises to perform multiple task preferences simultaneously, which represents the common cognition of team members (Bluedorn et al., 1999; O'Loughlin, 2016). On the other hand, Rebetez et al. (2016) found that individuals would adjust the task progress status through their existing resources and push the task toward the expected. But for individuals who need to participate in multiple tasks at the same time, their remaining time resources are less, their self-regulation ability is limited, and they cannot effectively adjust the task progress (Ahmad and Saud, 2016; Rebetez et al., 2016). Therefore, it may cause the task to fail to be complete successfully. Asghar et al. (2018) have found that individuals who participate in multiple tasks simultaneously have low time consciousness, which may also cause individuals to postpone completing the planned task. According to the theory of resource conservation, focusing on the group and individual resources gives a precise framework for understanding the effect of emotion and performance assessment. The first priority of humans, according to the conservation of resource (COR) theory, is to build, defend, and foster additional resources in order to protect social relationships, which in turn protect self (Alvaro et al., 2010). Based on theory, a model is offered that helps in engaging in proper behavior by preventing resource loss, gaining new resources, and maintaining existing ones (Hobfoll, 2011). Based on the above analysis, this study proposes the following hypothesis:

H1: The team level polychronicity is a positive effect on the member procrastination behavior.

### Multitasking and Members Work Overload

The workload is a term that relates to the complexity of systems of work that employees should complete (Bolino and Turnley, 2005). This study concentrates on quantitative stress since it has garnered extensive attention in the restoration domain (DeArmond et al., 2014). In this perspective, we regard workload as a source of job stress that is likely to affect task detachment negatively. Matthews et al. (2014) stated that individuals with a strong workload or high expectations might work more from the household. Furthermore, they may predict a big schedule at work, which may make disengagement more challenging (Paden and Stell, 1997; Wan et al., 2014). Moreover, a heavy workload or a greater amount of expectations may be associated with increased types of depression activation, making it more difficult to unwind and forget about work (Dux et al., 2009). Strong empirical evidence suggests that increased effort is associated with high procrastination (van Eerde, 2016).

According to the stressor paradigm, detachment of task is most likely related to procrastination. Our primary indicator of strain in this study is procrastination (Dukes et al., 2013). There is relatively limited research available that task detachment is correlated with greater fatigue and with other measures of employees procrastination behavior (Gupta et al., 2012). For example, discovered a negative correlation between even detachment and future weariness. Asghar et al. (2020) showed moderately high unfavorable associations between detachment and psychosomatic problems and between increased procrastinating.

Moreover, the multitasking trend of the team represents the work requirements of the team on the members, but this trend does not increase the working time of the members. Therefore, as the multitasking trend of the team increases, the work requirements of the team for members gradually exceed the working hours of employees, and members have demanding perceptions, namely, the role of work overload. According to the viewpoint of COR theory, the fewer the resources, the greater the fear of losing them (Alvaro et al., 2010). On the contrary, those who already have a huge amount of resources, are likely to acquire more. The loss of resources at the initial stage can have a negative impact on individuals and groups, demoralizing them. Even those with plenty of resources might be discouraged by ongoing resource loss, no matter how resilient they are (Hobfoll, 2012; Holmgrenn et al., 2017). To perform multiple tasks simultaneously, the time resources they have are also consumed and preserve resources. Therefore, they reduced their efforts, thereby delaying the completion of the original plan (O'Loughlin, 2016). In addition, task switching between different tasks has also sped up the perception of resource consumption, especially the conversion between different types of tasks (Stead et al., 2010). Therefore, as the multitasking trend goes down, the individual feels that the role is overloaded. To find a psychological balance, the effort on the goal may be reduced, and procrastination may occur (Holmgrenn et al., 2017). Ahmad and Saud (2016) have discovered that engaging in multiple tasks has changed the perception of the workload of an individual and then cause some negative effects.

H2: Work overload as a mediating role in the relationship between team multitasking trends of individuals and member procrastination behavior.

### The Moderating Role of Team Cohesiveness

Conservation resource theory also stated that individuals are trying to alleviate the perception of resource loss by reallocating time resources (Hobfoll, 2001). Alvaro et al. (2010) explain COR theory that social support is extremely important. People who have fewer resources at their disposal are more likely to feel burden and stress. People can contact a friend or family member, in this case, to obtain enough resources to ease the stressful work environment. This reallocation of time resources can be reflected in the arrangement of working hours, that is, work cohesiveness (Hobfoll, 2012). A group consists of two or more people who share common interests' objectives, and it through (Hobfoll, 2011). The majority of persons belong to groupings that can be categorized in a kind of circumstances. Groups can be classed in a variety of ways. For instance, they can be classified as friendship groups or task groups. Informally, a friendship group forms to meet the needs of members for security, esteem, and belongingness (Langfred, 1998; Brockman and Morgan, 2006). A friendship network among coworkers may develop over time.

In addition, electronic enhancements are being made to friendship groups. MySpace, Facebook, Twitter, Blogger, YouTube, and instant messaging are all examples of technological tools used to create and maintain friendship networks (Aral et al., 2007). Leaders form task groups to fulfill specific organizational objectives. A single group may serve both companionship and task functions (Dux et al., 2009). Several variables affect team cohesiveness, including member interaction, team size, stringent admission requirements, team success, and external competition and difficulties. Cohesiveness typically increases with the amount of time team members spend together. Team cohesion is maximized when teams are kept small enough to execute the duties yet large enough to collaborate (Li et al., 2017). When teams have a rigorous admissions process, they tend to be more cohesive. Cohesiveness increases in direct proportion to the success of the team. When members encounter external competition or a difficult goal, team cohesiveness tends to strengthen (Holmgrenn et al., 2017). Matt Graham and Jones (2019) have suggested that carrying out multiple tasks at the same time may produce positive results and may also lead to negative results, and it is critical to identify the time conditions during which they act.

Thus far, we have emphasized the importance of cohesion as a good characteristic. It is possible members of highly group cohesive are more engaged in activities of their team, are absent less frequently, have a low turnover rate (Wendt et al., 2009), meet a variety of individual needs, including emotional and social identity needs, and are occasionally exceptionally productive (Garg et al., 2018). While cohesive teams are beneficial to their members, they may or may not be beneficial to the organization as a whole (Peng and Kamil, 2018). Liu et al. (2019) have been revealed that the failure of this adjustment will increase the resource consumption of an individual and increase the role of overload. Zhijie et al. (2019a) have believed that employees who need to perform multiple tasks simultaneously desire an environment where they can freely arrange working hours. Team cohesiveness fosters a high degree of motivation and dedication to the team, which results in increased team performance (Matthews et al., 2014). Therefore, it can be inferred that there is a difference in the impact of team multitasking on the role of overload (Yile, 2020; Gull et al., 2021). For employees working in a team cohesiveness environment, team multitasking tends to affect role overload. Positive effects are mitigated for employees working in a less autonomous environment. Hence, we propose the hypothesis.

H3: Team level cohesiveness is a moderating role and weakens the relationship between team multitasking trends and work overload.

Based on the H2 and H3 hypotheses mentioned above, this study proposes that the mediation effect of work overload is controlled by team cohesiveness. Precisely, when individuals work in teams with high cohesiveness, they can flexibly determine working hours and working methods, freely redistribute available time resources, balance the relationship between work requirements and individual time resources, and reduce multitasking trends to overload character roles (O'Loughlin, 2016). As a result, the efforts of employees to reduce resources for resource conservation purposes will also decrease, and the frequency of procrastination will decrease. Moreover, when individuals work in a team with a high level of cohesiveness, they can only obey the workflow in the team, and it is difficult to redistribute time resources flexibly (Kokoç, 2021). This limitation has instead exacerbated the imbalance between work requirements and individual time resources. Furthermore, intelligence enhances the perception of character overload. Therefore, employees are more likely to threaten resource consumption and are more likely to reduce their efforts to the goal to seek psychological balance for resource conservation, increasing the frequency of procrastination, based on this, this article proposes hypotheses.

## Methodology

### Data Collection and Sample Source

This study employed a cross-sectional design and a quantitative methodology, as data were acquired *via* questionnaires. This questionnaire was distributed by hand and was coded to identify people who worked in the same workgroup. ICTs employees report the questionnaire items based on their observations, and they have been informed that the content of the questionnaire is completely secret. It was exclusively used for scientific research purposes to ensure that respondents could react confidently. The sample consisted of 28 ICT enterprises in Punjab, Pakistan. A total of 254 employees representing 88 work teams participated in the survey. After excluding three groups with invalid questions, a total of 231 valid questionnaires were obtained, encompassing 76 members of working teams in 27 ICTs industries. All factors were quantified using a predeveloped scale. Men accounted for 51.1% of all valid samples improved, while women accounted for 48.9%; the average age is 30 years; education level is primarily concentrated at the bachelor's degree level and above, and the average working life in this type of organization is 2 years.

### Measurement Scale

The scales of measurement utilized in this investigation are already developed. To avoid confusion during the formal survey, the questionnaire entries have been modified to ensure the reliability and validity of the questionnaire. The multitasking tendencies of the team were quantified using an eight-item scale (Bluedorn et al., 1999). In this study, the internal consistency coefficient is set to 0.909. The procrastinating behavior was assessed using an 11-item pure procrastination scale developed by researchers (Svartdal and Steel, 2017). In this investigation, the internal consistency coefficient is 0.896. The work overload scale is composed of three items. In this study, the four-item scale used to assess team cohesiveness has an internal consistency coefficient of 0.840. All items were rated on a five-point Likert scale and demographic variables, such as age, gender, and education were included.

### Analysis and Results

The statistical software, Mplus, was used to do cross-layer linear regression analysis on the data. First, while multitasking and team cohesiveness are group-level variables, the assessments of this study are based on individual-level accounts. As a result, a pooling test and the combining method were used to show that the mean value of the formative evaluation was employed as the observation value of the group. Specifically, (1) the intraclass correlation (ICC) of the multitasking tendency of the team is 0.38, (2) the ICC of the cohesiveness of the team is 0.31, the ICC (2) of the cohesiveness of the team is 0.57, and the rw_g_ mean is 0.71; all of these values satisfy the proposition of convergence, indicating that the average value of the multitasking tendency of the team and team cohesiveness at the individual level can be used as the observation value of the group. Second, typical single-level linear regression does not provide adequate explanations. Single-level regression models that do not distinguish between within-group and between-group variation may produce problematic findings (Muthén and Muthén, 2017). The primary analysis method in this study was a multilayer linear model. The distinctions within the grouping strengthen the reliability of conclusions drawn from the distinctions between groups. Finally, this study was corrected utilizing cross-layer regression analysis by the recommendations. According to, the Monte Carlo Bootstrapping test was included during the mediating effect in this section to ensure the validity of the conclusions.

## Data Analysis and Results

### Common Method Bias

The questionnaires were distributed and improved only one time in this study, and questionnaires were filled by the survey respondents separately, which may be affected by homogeneous variance. The results of one-way test show that the variance of the first factor is 31.553%. Therefore, it can be considered that the data used in this study does not have serious common method deviation problems and have certain reliability.

Confirmatory factor analysis (CFA) can be used to ascertain the distinct validity of variables. The four factors models used in this article include four factors, three factors, two factors, and other combinations, and each factor is subjected to CFA separately. The results of several models are shown in Table 1. Among all, the four-factor model (Model 1) had the best fit. Thus, if the terms correspond to four variables and the variables have appropriate discriminant validity for further regression analysis, the challenges associated with measuring variables can be evaluated.

**Table 1:** Different validity analysis of variables.

**Model**	**Factors**	**χ^2^**	**Df**	**Δχ^2^/Δ_df_**	**CFI**	**TLI**	**RMSE**	**SRMAR**
Model 1	4 factors	436.064	278		0.94	0.93	0.068	0.062
Model 2	3 factors	9109.945	258	137.44**	0.87	0.84	0.083	0.081
Model 3	2 factors	1428.883	276	246.70**	0.69	0.56	0.142	0.171
Model 4	1 factor	2010.836	272	224.25**	0.48	0.44	0.162	0.162

** Indicates that 0.01 level of significance and *Means 0.05 levels of significance.

As shown in Table 2, the correlations of the variables involved in this study are listed. Multitasking trend perception, the role of overload, and procrastination behavior all exist significant positive correlation, which is consistent with the research hypothesis. However, its interaction and strength need to be verified by subsequent analyses.

**Table 2:** Correlation analysis of variables.

**Variable**	**Mean**	**SD**	**1**	**2**	**3**	**4**	**5**	**6**	**7**	**8**
Gender	1.87	0.401	1							
Age	29.98	2.891	0.023	1						
Educations	2.01	2.713	0.028	−0.019	1					
Experiences	1.97	1.017	0.019	0.324**	−0.004*	1				
Multitasking	2.78	1.787	−0.021	−0.106*	0.051*	−0.031*	1			
Work overload	1.68	2.104	−0.104	−0.132*	0.020*	−0.023*	0.480**	1		
Team cohesiveness	2.38	1.00	0.036	−0.032*	0.051	−0.006*	0.303*	−0.037*	1	
Procrastination behavior	3.71	0.837	0.052	−0.089*	−0.041*	−0.048*	0.410**	0.417**	−0.221**	1

** *0.01 level is significantly related*.

* *0.05 levels are significantly correlated*.

This study uses a multilayer linear regression model for hypothesis testing. First, the main effect of mediating work overload and procrastination behavior acts as a zero-model test for the dependent variable procrastination behavior. The results show that, without adding any variables, the persistent behavior of the intergroup difference is 0.249 (*p* < 0.01); the variance between groups for the role of work overload is 0.496 (*p* < 0.01). This shows that procrastination behavior and work overload are adequate differences between groups that can be tested by subsequent cross-layer regression (Table 3).

**Table 3:** Direct and indirect effects.

**Dependent variable**	**Work overload**		**Procrastination behavior**		
	**Step 1**	**Step 2**	**Step 3**	**Step 4**	**Step 5**
**Level - 1**					
Work overload					0.410**
**Level- 2**					
Multitasking		0.598**		0.420*	0.097
Work overload					0.396**
σ^2^	0.577	0.576	0.683	0.445	0.398
τ_00_	0.631**	0.503**	0.241**	0.336**	0.206**
*R*^2^_level−1_					22.01%
*R*^2^_level−1_		23.88%		14.98%	27.96%

** *0.01 level is significantly related*.

* *0.05 levels are significant*.

First, Model 2 and Model 4 show that there are many teams whose substantial tasks tend to predict the work overload at the individual level positively (γ = 0.608, *p* < 0.01) and procrastination behavior (γ = 0.350, *p* < 0.01), Hypothesis 1 is significant. Second, Model 5 shows that the role of the individual level is addressed according to the mean value (γ = 0.383, *p* < 0.01) and group-level roles, the mean values of the loaded groups (γ = 0.416, *p* < 0.01) are all significant. Forward-looking procrastination behavior, but the team is multitasking positively predicting the trend (γ = 0.098, *p* > 0.05), is no longer significant. It is contingent work overloaded to the full mediation effect H2 supported; Hypothesis 2 is significant. Finally, Model 2 states that team multitasking tends to explain role overload, 11% variance between groups; Model 4 states that team multitasking tends to explain procrastination, 81% between-group variance; Model 5 states that work overload is explained at the individual-level delayed behavior, 21.71% of the variance within the group, mean work overload at the group-level explained procrastination, 28.16% variance between groups.

**Figure 1:**
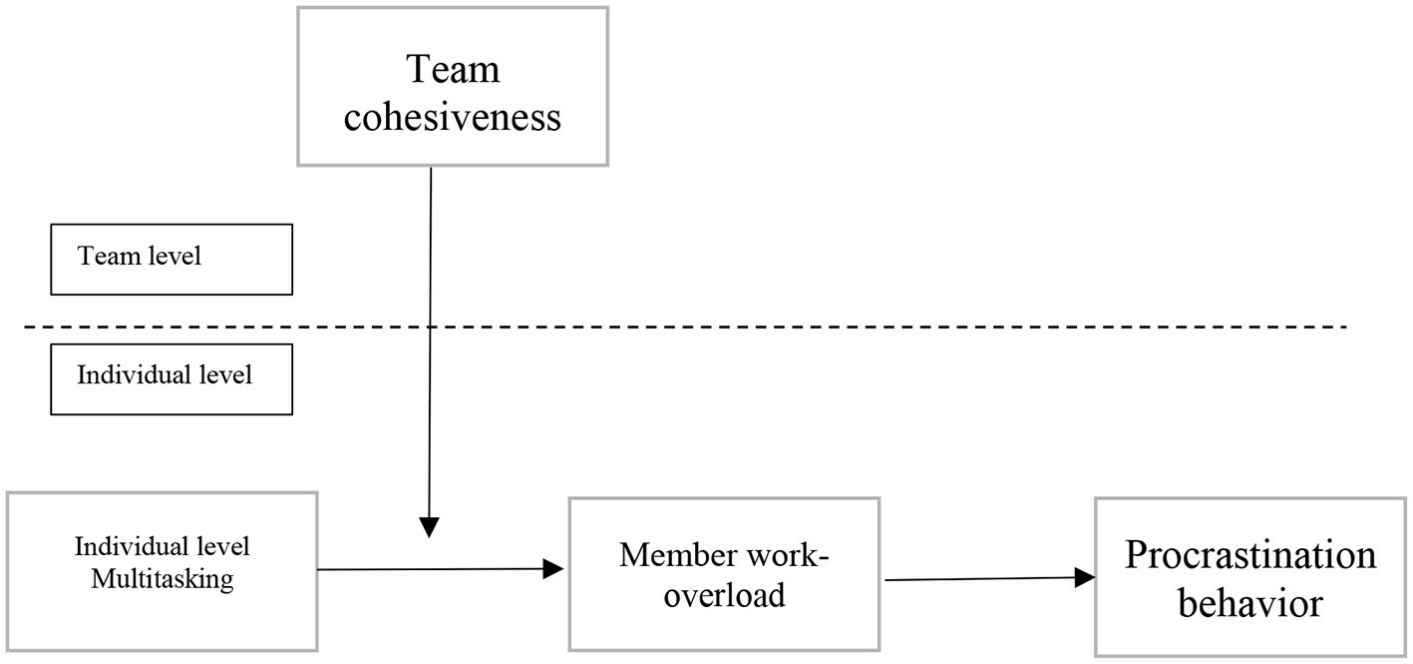
Multilevel modeling.

Table 4 shows the moderating effect of team cohesiveness, compared with Model 6, and team multitasking trend and team cohesiveness interaction, the term has a significant negative impact on role overload (γ = −0.467, *p* < 0.05), and this interactive term explains role overload with 9.79% between groups as poor. In order to show the regulation effect instinctively, the regulation effect diagram shown in Figure 1 is drawn from a multitasking scenario when team time cohesiveness is low.

**Table 4:** Moderating effect of team cohesiveness.

**Dependent variable**	**Work of overload**		**Procrastination behavior**	
	**Step 6**	**Step 7**	**Step 8**	**Step 9**
**Level - 1**				
Work overload				0.382**
**Level - 2**				
Multitasking	0.633**	0.674**	0.420**	0.198
Team cohesiveness	−0.189	−0.152	−0.266**	−0.216*
Cross-level interaction		−0.467*	−0.349**	−0.190
Multitasking × Team cohesiveness				0.330*
σ^2^	0.676	0.676	o.585	0.485
τ_00_	0.388**	0.350**	0.185**	0.147**
*R*^2^_level−1_				17.09%
*R*^2^_level−1_		9.79%		20.54%

** *0.01 level is significantly related*.

* *0.05 levels are significant*.

Multitasking trends have stronger effects on work overload, and when team cohesiveness is higher, team multitasking tends to have less effect on work overload H3 is proved. To verify the use of mediation effect, we performed a Monte Carlo test, and the test results showed that within 90% CI, indirectly, the impact was significantly negatively measured [90% CI (−0.473, −0.02)], accordingly, Hypothesis 4 is supported.

## Discussion and Conclusions

The purpose of this study was to examine the influence of ICT-encouraged multitasking on procrastinating behavior of Pakistan members. Moreover, multitasking with ICTs is encouraged among team members. Nevertheless, multitasking is not necessarily detrimental to cognitive performance and may even be beneficial. Procrastination is an individual behavior with time characteristics, and the multitasking tendency reflects the preferences of the team on time usage and has certain time characteristics (Kokoç, 2021). Group team orientation can influence many studies that have confirmed the behavior of individual employees. Therefore, in this study, the multitasking trend of the team and individual delays in connecting, emphasize the concern of time-related constructs and fill a gap in the field of multitasking trends affecting delayed behavior.

This study extends the research on the time use preferences of the team to the realm of individual behavior ICT industry's in Pakistan. This study discovered work overload as a mediating role in the relationship between group-level polychronicity and member procrastination behavior. In addition, the moderating role of team cohesiveness and group-level polychronicity and member workload. With scholars focusing their attention on the properties of time in recent years, the theory of resource conservation has increasingly been applied to this field (Holmgrenn et al., 2017).

In this study, group-level multitasking increases the work overload of employees, which will cause members procrastination behavior that time resources are fast rapid consumption, there is a huge threat. Therefore, a stressful situation occurs when there is a threat of resource loss, actual resource loss, or the failure to obtain resources despite investment and people spend their resources to avoid potential resource loss (Park et al., 2018). This study reveals multitasking of team simultaneously throwing light on the mechanism of individual procrastination. The scope of application of resource conservation theory has been extended to the field of organizational psychology.

**Figure 2:**
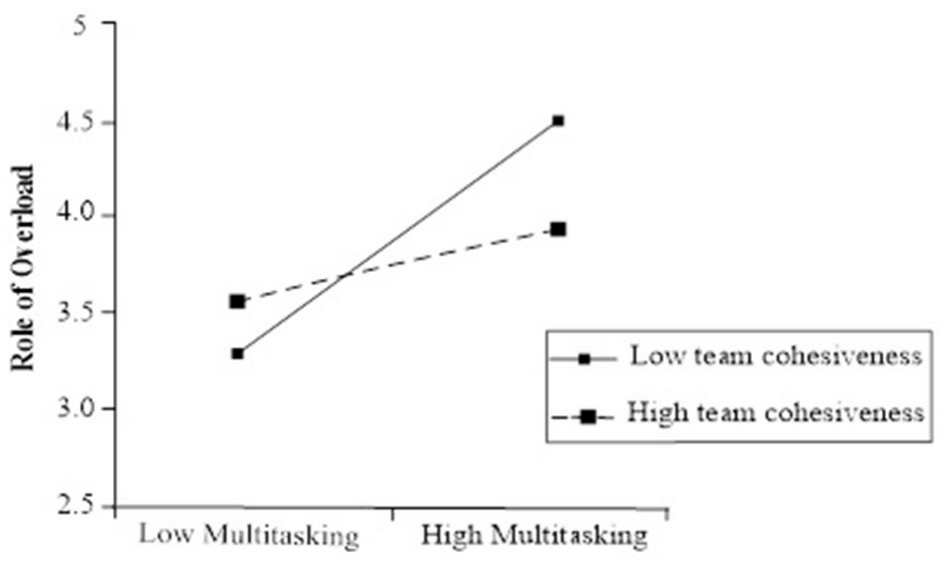
Team cohesiveness.

Finally, this study finds the moderating role of team cohesiveness and improves the role of team multitasking trends on individual procrastination. Team cohesiveness is an important boundary condition to alleviate the negative effects of job requirements. The conservation resources theory states that individuals can redistribute time and resources. The COR theory states that individuals have an inborn quality that is activated during stress to recover lost resources. The basic principle of COR theory is that people tend to maintain, gain, protect, and foster what they value the most. Resources are the things that people value the most because resources are useful in achieving desired outcomes (Hobfoll et al., 2018). The source is to alleviate the sense of resource consumption. The premise of this redistribution is cohesiveness, and team cohesiveness, as a change, better reflects the true state of cohesiveness measurements, but very few studies focus on it. In response to the call to focus on team cohesiveness, this study treats team cohesiveness as a group level, the moderator variables have been included in the study model. It was found that team cohesiveness can reduce the role of overload caused by the multitasking tendency of the team, further debilitating the entire process of “team multitasking tendency perception through work overload and thus procrastination behavior.” The results make up for previous research the lack of attention to the cohesiveness regulation role of the team has enriched the boundary conditions of the multitasking tendency of the team to influence the role and helps to understand how to develop better and play the positive role of the multitasking trends of the team (Figure 2).

### Practical Implications

Current research on team multitasking and ICTs has examined the members' workload and the relationship between group-level polychronicity and procrastination behavior at the individual level in this study. Several factors affect efficiency during group-level multitasking in ICTs, including the amount of time spent communicating, the number of communication events, interruptions, overlapping communications, the communication channel chosen, and the communication goal. According to others, team-level multitasking has little influence on the analytical ability or work overload of individuals as they focus on numerous jobs. Others doubt the feasibility of multitasking, i.e., functioning on multiple activities concurrently. While training in multitasking *via* ICTs or group-level multitasking has been shown to improve executive attention, neuroimaging studies have shown that the effects of training on executive attention are manifested in the tuning of the brain areas involved in the network and also in improved connectivity between areas *via* axon expansion and myelination. Interestingly, persons who prefer to engage in group-level multitasking do not appear to be particularly adept at task-switching or attention tasks, even when they are confident about their ability to perform these tasks successfully. This study found that group-level multitasking provides them enjoyment. Multitasking was positively correlated with the emotional satisfaction of University students, albeit at the cost of cognitive performance. Multitasking is necessary for certain professions and is an indisputable phenomenon in education and life. Multitasking can be an efficient use of time, a relatively manageable endeavor when required, or, when well-monitored or well-regulated, an effective tool in problem-solving. Furthermore, people, who are hyperconnected, in general report that they do not have problems attending to everyday tasks and interpersonal relationships.

The conclusion of this research has a certain guiding value for management daily. First, group-level multitasking may be a factor in the delayed behavior. While multitasking may have numerous benefits for a company, it may also work against its objectives. Managers should be acutely conscious of the tendency toward the “double-edged sword” effect of multitasking. One cause for the procrastination behavior of an individual is that he/she attempts to engage in various projects and distributes his/her time resources, resulting in a lack of resources required to accomplish the task and time wasted when the assignment was changed. As a result, the team of an organization cannot pursue additional tasks blindly in a short period.

When an organization and team leader care about the employees, they can reduce their role overload. The multitasking tendency perception of an individual will increase the level of procrastination. Therefore, how to relieve the work overload of an employee is a crucial problem. Managers in an organization can empower, provide support, set a reasonable workload, and other ways to balance the relationship between work requirements and work resources of employees. Mutual support between colleagues is also one of the ways to reduce the overload of an employee. By relieving the work overload, procrastination can be avoided.

Team cohesiveness better reflects the true situation of cohesion than individual coherency, and it is also closer to the institutional arrangements within the organization. Team cohesiveness can effectively alleviate team members' character's sense of work overload reduces procrastination. Therefore, an enterprise should fully authorize the team to exercise discretion when formulating a management system and workflow. The measures reduce rigid team workload and arrangements and allow employees to truly feel the real existence of cohesiveness, so that team members can improve their time control ability, and by reallocating time resources to balance the relationship between work requirements and work resources, reduce negative behavior. Multitasking at the group level is thought to be a component of ICT in terms of their services to software houses. We cannot underestimate the importance of social networks and ICT in today's globalization, particularly in the IT field of endeavor, but there is a need to provide a policy that assists in regulating the affairs by IT professionals and other stakeholders to reduce the level of procrastination among members; members engaged in social networking for purposes other than mental procrastination. Therefore, it is beneficial if the organization and team leader establish policies for workload reduction and performance enhancement.

### Limitations and Future Study

The limitations of this study are mainly as follows: First, this is based on cross-sectional data during the research. Future research will be done on longitudinal data and interview-based studies. This study-based questionnaire filled by individual employees may exist in the same source. This method creates a common basis in research. In the future, we will try to use multisource and multipoint measurement methods to reduce homogeneous method bias. We are performing more rigorous statistical tests, which may be another limitation.

Second, although this study found the mediating role of work overload and the moderating role of team cohesiveness, the mechanisms and boundary conditions under other theories cannot be ruled out. Future research considers introducing other mechanisms to enrich the multitasking trend and delay the mechanism of the relationship and boundary conditions. Finally, procrastination is a common outcome variable in the field of psychology. In the field of management, but considering the limitations of the questionnaire survey method, in future research, we can use experimental methods commonly used in psychology to develop, explore, and realize the integration of interdisciplinary research.

## Data Availability Statement

All subjects gave their informed consent for inclusion before they participated in the study. The study was conducted in accordance with the Declaration of Helsinki, and the protocol was approved by the School of Business and Tourism Management, Yunnan University, Kunming, China.

## Ethics Statement

Written informed consent was obtained from the individual(s) for the publication of any potentially identifiable images or data included in this article.

## Author Contributions

NG and MA: conceptualization, methodology, investigation, resources, and data collection. ZA and NG: software and validation. ZA and MA: formal analysis, writing, and original draft preparation. MA: writing, review, and editing. TX: funding. All authors contributed to the article and approved the submitted version.

## Funding

This work was supported by Yunnan Philosophy and Social Science Planning Funding Project (YB2018017) and Yunnan University Double Tops Construction Funding Project (C176240104).

## Conflict of Interest

The authors declare that the research was conducted in the absence of any commercial or financial relationships that could be construed as a potential conflict of interest.

## Publisher's Note

All claims expressed in this article are solely those of the authors and do not necessarily represent those of their affiliated organizations, or those of the publisher, the editors and the reviewers. Any product that may be evaluated in this article, or claim that may be made by its manufacturer, is not guaranteed or endorsed by the publisher.
